# Oral Cannabidiol for Seborrheic Dermatitis in Patients With Parkinson Disease: Randomized Clinical Trial

**DOI:** 10.2196/49965

**Published:** 2024-03-11

**Authors:** Isaac Weber, Caterina Zagona-Prizio, Torunn E Sivesind, Madeline Adelman, Mindy D Szeto, Ying Liu, Stefan H Sillau, Jacquelyn Bainbridge, Jost Klawitter, Cristina Sempio, Cory A Dunnick, Maureen A Leehey, Robert P Dellavalle

**Affiliations:** 1 Mercy Hopsital St. Louis St. Louis, MO United States; 2 Department of Dermatology University of Colorado School of Medicine Aurora, CO United States; 3 Department of Neurology University of Colorado School of Medicine Aurora, CO United States; 4 Dermatology Service Rocky Mountain Regional Veterans Affairs Medical Center Aurora, CO United States; 5 Colorado School of Public Health Aurora, CO United States

**Keywords:** cannabidiol, cannabis, CBD treatment, CBD, image, photograph, photographs, imaging, sebum, clinical trials, seborrheic dermatitis, Parkinson disease, clinical trial, RCT, randomized, controlled trial, drug response, SEDASI, drug, Parkinson, dermatitis, skin, dermatology, seborrheic dermatitis, treatment, outcome, cannabis, chi-square

## Abstract

**Background:**

Seborrheic dermatitis (SD) affects 18.6%-59% of persons with Parkinson disease (PD), and recent studies provide evidence that oral cannabidiol (CBD) therapy could reduce sebum production in addition to improving motor and psychiatric symptoms in PD. Therefore, oral CBD could be useful for improving symptoms of both commonly co-occurring conditions.

**Objective:**

This study investigates whether oral CBD therapy is associated with a decrease in SD severity in PD.

**Methods:**

Facial photographs were collected as a component of a randomized (1:1 CBD vs placebo), parallel, double-blind, placebo-controlled trial assessing the efficacy of a short-term 2.5 mg per kg per day oral sesame solution CBD-rich cannabis extract (formulated to 100 mg/mL CBD and 3.3 mg/mL THC) for reducing motor symptoms in PD. Participants took 1.25 mg per kg per day each morning for 4 ±1 days and then twice daily for 10 ±4 days. Reviewers analyzed the photographs independently and provided a severity ranking based on the Seborrheic Dermatitis Area and Severity Index (SEDASI) scale. Baseline demographic and disease characteristics, as well as posttreatment SEDASI averages and the presence of SD, were analyzed with 2-tailed *t* tests and Pearson *χ*^2^ tests. SEDASI was analyzed with longitudinal regression, and SD was analyzed with generalized estimating equations.

**Results:**

A total of 27 participants received a placebo and 26 received CBD for 16 days. SD severity was low in both groups at baseline, and there was no treatment effect. The risk ratio for patients receiving CBD, post versus pre, was 0.69 (95% CI 0.41-1.18; *P*=.15), compared to 1.20 (95% CI 0.88-1.65; *P*=.26) for the patients receiving the placebo. The within-group pre-post change was not statistically significant for either group, but they differed from each other (*P*=.07) because there was an estimated improvement for the CBD group and an estimated worsening for the placebo group.

**Conclusions:**

This study does not provide solid evidence that oral CBD therapy reduces the presence of SD among patients with PD. While this study was sufficiently powered to detect the primary outcome (efficacy of CBD on PD motor symptoms), it was underpowered for the secondary outcomes of detecting changes in the presence and severity of SD. Multiple mechanisms exist through which CBD can exert beneficial effects on SD pathogenesis. Larger studies, including participants with increased disease severity and longer treatment periods, may better elucidate treatment effects and are needed to determine CBD’s true efficacy for affecting SD severity.

**Trial Registration:**

ClinicalTrials.gov NCT03582137; https://clinicaltrials.gov/ct2/show/NCT03582137

## Introduction

Seborrheic dermatitis (SD) is related to increased sebum production and an inflammatory response to cutaneous *Malassezia*, and it affects 18.6%-59% of persons with Parkinson disease (PD) [1,2]. The mechanism connecting these two pathologies is not entirely clear; however, increasing evidence suggests a direct role of *Malassezia* in the pathogenesis of PD [2]. Other proposed mechanisms include gene polymorphisms leucine-rich repeat kinase 2 (LRRK2), glucocerebrosidase (GBA), and phosphatase and tensin homolog–induced kinase 1 (PINK1); these have been shown to play a role in lipid regulation and increase the risk for PD in affected individuals [2]. Traditional first-line SD treatment relies on topical antifungals or anti-inflammatories, with systemic therapies reserved for recalcitrant or severe cases, which become more common in patients with immune dysfunction [2]. These systemic therapies, such as oral terbinafine and itraconazole, have numerous side effects, including hepatotoxicity and interactions with concomitant medications [3].

Delta-9-tetrahydrocannabinol (THC) induces a “high,” psychosis, cognitive dysfunction, and anxiety, while cannabidiol (CBD) has been reported to reduce sebum production and improve motor and psychiatric symptoms in PD [2-9]. CBD is likely safer than THC; however, some individuals with PD report the use of both and claim greater benefits from THC [4,10]. After oral consumption, THC travels to the liver, where the majority is eliminated or metabolized into other molecules by cytochrome P450 2C (CYP2C) and CYP3A [11]. The bioavailability of ingested THC is between 4% and 12% [11]. The pharmacokinetics of CBD are complex, and the bioavailability of oral CBD is estimated to be only 6% [11]. In general, the most abundant metabolites of CBD are hydroxylated 7-COOH (7-carboxy) derivatives that are excreted either intact or as glucuronide conjugates [12].

The use of CBD on human sebocytes has been shown to reduce sebaceous gland proliferation and induce anti-inflammatory changes [13]. However, few studies exist evaluating oral CBD’s effect on SD severity. CBD may be beneficial in both PD and SD, and research is needed to define what cannabinoids and doses are useful in both conditions. Based on current literature, an oral formulation with the following combination was pursued: greater CBD than delta-9-THC, with between 150 and 1000 mg CBD, and <10 mg THC daily [4-9,14,15]. This study investigates whether oral CBD therapy is associated with a decrease in SD severity in PD.

## Methods

### Overview

Facial photographs were collected as a component of a randomized, parallel, double-blind, placebo-controlled trial assessing the efficacy of a short-term 2.5 mg per kg per day of oral sesame solution CBD-rich cannabis extract for reducing motor symptoms in PD. The study drug, supplied by the National Institute of Drug Abuse as a frozen extract, was formulated to a 100 mg per mL CBD and 3.3 mg per mL THC sesame oil solution by a PharmD team. The placebo was compounded with *United States Pharmacopeia* (*USP*)–grade sesame oil, food coloring, and strawberry extract. ElSohly Laboratories, Inc performed stability, potency, and microbial analyses.

Participants took 1.25 mg per kg per day each morning (approximately 1 mL) for 4 ±1 days and then twice daily for 10 ±4 days. To test short-term use, the duration of time on the study drug was at least the minimum time needed for CBD to be at a steady state concentration. The half-life of oral CBD and oral THC is approximately 2 days and 4 hours, respectively [16]. To facilitate the interpretation of effects, cannabinoid plasma levels were documented at the final dose visit.

Eligibility criteria were defined by adults 40-85 years of age with idiopathic PD participating in the above trial and who had concurrent SD. Data were collected from the University of Colorado Hospital from September 2018 to January 2022. The sample size was determined by the number of patients in the trial meeting eligibility criteria. Eligible candidates were randomized 1:1 to the study drug or placebo by a computer-generated randomization schedule, stratified by age (45-60 vs 61-85 years) and modified Hoehn and Yahr scale score (1-2.5 vs 3-5) into blocks of four, with 2 participants per block being assigned to each treatment group [17].

The statistician (author SHS) and the PharmD team were the only unblinded study staff. The statistician generated the random allocation sequence. The statistician notified the lead PharmD (author JB) via encrypted email of the allocation assignment. The appropriate study drug was prepared by the PharmD team within days of the baseline visit. Blinded study staff enrolled participants and provided them with the study drug.

Despite best efforts, the placebo was slightly different in appearance and odor, so procedures were developed to optimize the preservation of the blinding. The design of the study was changed from crossover to parallel; the study drug for each participant was prepared in a brown opaque bottle that was placed into a “masking envelope,” a thick brown postage envelope with plastic bubble wrap lining to obscure odor, and the study drug was administered in a closed, vented room that removes the odor of cannabis within 4 minutes. Blinded study staff did not enter for at least 10 minutes. Further, the study drug was transported by the participants to their homes and the clinic in the masking envelope.

Deidentified photographs pre- and posttreatment were provided to two board-certified dermatologists to assess along with reference images external to the study. Reviewers analyzed the photographs independently, providing a severity ranking based on the Seborrheic Dermatitis Area and Severity Index (SEDASI) scale, a quantitative grading instrument [18]. Severity scores were averaged between reviewers for the final SEDASI score, and reviewers determined whether each participant’s SD had improved, worsened, or was unchanged. The possible range of scoring is 0 to 60, with 60 being the most severe.

Baseline demographic and disease characteristics were compared between treatment groups with 2-tailed *t* tests and Pearson *χ*^2^ or Fisher exact association tests. The presence of SD was analyzed longitudinally with generalized estimating equations relative risk models. Covariates of gender, age, and log-scaled PD disease duration were considered as time-interacting covariates. The final CBD blood level was also considered as an adjusting covariate for the posttreatment time point in the CBD group. SEDASI was analyzed with longitudinal regression. The change in SEDASI averages was analyzed with change scores, paired *t* tests for within-group changes, and a 2-sample *t* test on the change scores for the difference between groups. The CONSORT (Consolidated Standards of Reporting Trials) reporting guidelines were used and followed in the reporting of this trial [19].

### Ethical Considerations

The Colorado Multiple Institutional Review Board granted ethical approval (17-2318). All participants provided written informed consent. An independent data and safety monitoring board provided oversight.

## Results

A total of 27 participants received a placebo and 26 received CBD for 16 days; cannabinoid plasma levels are shown in Table 1. Baseline participant characteristics were similar between groups for most variables, although the study drug group trended toward longer disease duration (*P*=.07) and higher total Movement Disorders Society Modified Unified Parkinson’s Disease Rating Scale (MDS-UPDRS) score (*P*=.08), but this was not significant. There were no effects on orthostatic blood pressure, heart rate, or temperature, comparing before the first study medication dose to the final dose and comparing before a dose to 1-3 hours afterward. There were also no notable changes in blood laboratory studies, including liver tests. The study drug was tolerated with no unexpected and serious adverse effects and no significant dermatological adverse events. SD severity was low in both groups at baseline, and there was no treatment effect, as shown in Table 2. Generalized estimating equation regression analysis, where final blood level of CBD was included as an explanatory variable and for which there were 26 patients receiving CBD and 27 patients receiving placebo with data, revealed that CBD treatment trended toward reducing the presence of SD compared with the placebo (*P*=.07 at the mean CBD final blood level of 49.29 ng/mL) because there was an estimated improvement for the CBD group and an estimated worsening for the placebo group, but this finding did not reach statistical significance. The estimated prevalence post-pre ratio of SD in the CBD group was 0.69 (95% CI 0.41-1.18; *P*=.15), compared to 1.20 (95% CI 0.88-1.65; *P*=.26) in the placebo group.

**Table 1:** Demographic characteristics and presence of seborrheic dermatitis for patients with Parkinson disease in oral cannabidiol (CBD) and placebo treatment groups.

Characteristic			CBD (n=26)		Placebo (n=27)		*P* value^a^
Age (years), mean (SE; SD)			70.6 (1.2; 6.3)		68.7 (1.4; 7.5)		.34
**Gender, n (%)**							.33
	Female	10 (38)		7 (26)			
	Male	16 (62)		20 (74)			
**Race**							>.99
	Asian	0 (0)		1 (4)			
	White	26 (100)		26 (96)			
**Ethnicity**							>.99
	Hispanic or Latino	1 (4)		0 (0)			
	Not Hispanic or Latino	25 (96)		28 (96)			
	Not reported	0 (0)		1 (4)			
**Employment**							>.99
	Disabled, permanently or temporarily	1 (4)		1 (4)			
	Retired	20 (77)		21 (78)			
	Working now	4 (15)		4 (15)			
	Partly retired	1 (4)		0 (0)			
	Retired, still involved in business	0 (0)		1 (4)			
**Marital status**							>.99
	Divorced	4 (15)		4 (15)			
	Living with partner	0 (0)		1 (4)			
	Married	21 (81)		21 (78)			
	Widowed	1 (4)		1 (4)			
Duration of PD^b^ (years), mean (SE; SD)			6.6 (1.3; 6.8)		4.6 (0.8; 4.0)		.19
**Dosing^c^, mean (SD; SE)**							N/A^d^
	Final CBD dose (mg/day)	187.50 (56.68; 11.12)		N/A			
	Final THC^e^ dose (mg/day)	6.28 (1.90; 0.37)		N/A			
	CBD level at final dose visit (ng/mL)	49.29 (32.85; 6.44)		0.00 (0.00; 0.00)			
	THC level at final dose visit (ng/mL)	0.85 (0.91; 0.18)		0.00 (0.00; 0.00)			
Time on study drug (days), mean (SD; SE)			15.5 (1.8; 0.3)		16.2 (1.6; 0.3)		.15

^a^Two-tailed *t* tests and Pearson *χ*^2^ or Fisher exact association tests.

^b^PD: Parkinson disease.

^c^Blood levels reflect 26 participants in the CBD group and 17 in the placebo group. Blood levels were not obtained for 3 participants in the CBD group and 2 in the placebo group.

^d^N/A: not applicable.

^e^THC: tetrahydrocannabinol.

**Table 2:** Results for patients with Parkinson disease in oral cannabidiol (CBD) and placebo treatment groups.

			Pretreatment		Posttreatment		*P* value
**Presence of seborrheic dermatitis** ^a^ **, n (%; 95% CI)**							
	CBD	12 (46.2; 30.5-69.9)		9 (34.6; 20.4-58.7)		.26	
	Placebo	15 (55.6; 39.7-77.9)		18 (66.7; 51.1-87.0)		.26	
	Treatment effect	N/A^b^		N/A		.12	
**SEDASI** ^c^ **average** ^d^ **, mean (95% CI; SD)**							
	CBD	3.63 (1.41-5.86; 5.50)		3.79 (1.38-6.20; 5.96)		.81	
	Placebo	5.39 (2.75-8.03; 6.68)		4.65 (2.76-6.54; 4.77)		.35	
	Treatment effect	N/A		N/A		.38	

^a^Presence of seborrheic dermatitis indicates patients exhibiting any signs of seborrheic dermatitis after assessing the final SEDASI score. Numbers calculated for generalized estimating equation model with log link (ie, relative risk model with repeated measures).

^b^N/A: not applicable.

^c^SEDASI: Seborrheic Dermatitis Area and Severity Index.

^d^SEDASI average is calculated by averaging the two scores assigned by independent reviewers to each patient.

## Discussion

### Principal Findings

This study does not provide solid evidence that oral CBD therapy reduces the presence of SD among patients with PD. While this study was sufficiently powered to detect the primary outcome (efficacy of CBD on PD motor symptoms), it was underpowered for these secondary outcomes of detecting changes in the presence and severity of SD. CBD has shown significant promise in improving SD in a topical form; however, no current literature exists to evaluate its effect when taken orally [20].

The pathophysiology of SD is still not entirely understood, but the colonization of *Malassezia* is strongly associated with the condition [1]. *Malassezia* is found on sebum-rich skin, and its metabolites have been shown to induce inflammation and stimulate keratinocyte differentiation, resulting in dysfunction in the stratum corneum, a disruption of the epidermal barrier, and perpetuation of an inflammatory response, leading to a cycle of more skin barrier disruption and the clinical manifestations of SD [21-23].

CBD possesses the ability to inhibit the lipogenic action of arachidonic acid, linoleic acid, and testosterone in human sebocytes; in addition, it has been shown to suppress sebocyte proliferation via ion channel activation [13,24]. CBD also possesses anti-inflammatory properties through the inhibition of nuclear factor kappa B (NF-κB) and signaling and upregulation of tribbles pseudokinase 3 (TRIB3) [13]. These mechanisms help explain its success in improving SD symptoms with topical therapy and provide a strong impetus for further study with oral CBD and SD.

### Limitations

Limitations include study drug availability constraints, limiting the time participants were on the study drug. A 16-day treatment period may not have been long enough to achieve maximal clinical benefit. Additionally, although the prevalence of SD for study participants was similar to existing estimates, low levels of disease severity in the cohort, both pre- and posttreatment, posed a challenge for assigning scores and may have impacted the reviewers’ ability to detect change. Possible confounders include participants’ concurrent topical medication use, which also hinders the interpretation of the findings.

### Conclusion

Larger studies, including participants with increased disease severity and with longer treatment periods, may better elucidate treatment effects and are needed to determine CBD’s true efficacy for SD severity. Oral CBD has shown promise in improving Parkinsonian symptoms; therefore, if future studies can elicit improvement in SD as well, it could act as a useful adjunct for patients struggling with PD to improve both neurologic and common cutaneous symptoms.

## Appendix

Multimedia Appendix 1 CONSORT (Consolidated Standards for Reporting Trials) checklist.

## Abbreviations

7-COOH: 7-carboxy
CBD: cannabidiol
CONSORT: Consolidated Standards of Reporting Trials
CYP2C: cytochrome P450 2C
GBA: glucocerebrosidase
LRRK2: leucine-rich repeat kinase 2
MDS-UPDRS: Movement Disorders Society Modified Unified Parkinson’s Disease Rating Scale
NF-κB: nuclear factor kappa B
PD: Parkinson disease
PINK1: phosphatase and tensin homolog–induced kinase 1
SD: seborrheic dermatitis
SEDASI: Seborrheic Dermatitis Area and Severity Index
THC: tetrahydrocannabinol
TRIB3: tribbles pseudokinase 3
USP: United States Pharmacopeia
